# Green and Ultrafast Preparation of TiO_2_ Aerogel Assisted by Mechanical Ball Milling

**DOI:** 10.3390/molecules31152690

**Published:** 2026-08-02

**Authors:** Nai Liu, Jingyi Chen, Dongzhi Zhu, Kao Chen, Qingge Feng

**Affiliations:** 1 School of Resources, Environment and Materials, Guangxi University, Nanning 530004, China; liunai_96@163.com; 2 School of Materials and Environment, Guangxi Key Laboratory of Advanced Structural Materials and Carbon Neutralization, Guangxi Engineering Research Center for Advanced Materials and Intelligent Manufacturing, Guangxi Minzu University, Nanning 530105, China; cjy028888@163.com (J.C.); 20230021@gxmzu.edu.cn (D.Z.); 3 College of Civil Engineering and Architecture, Guangxi University, Nanning 530004, China

**Keywords:** green and ultrafast synthesis, TiO_2_ aerogel, colloidal processing, mechanical force-assisted method, porous materials

## Abstract

TiO_2_ aerogel is a uniquely promising photocatalyst that has been widely applied in water environment remediation. Despite continuous advances in its synthesis methods, bottlenecks such as long preparation cycles, large organic solvent consumption, and high production costs still restrict its large-scale application. This work presents a mechanical force-assisted (MFA) strategy for green and ultrafast TiO_2_ aerogel preparation, with process intensification as its core innovation. The total preparation time is reduced by 60%, and n-hexane consumption is decreased by 25%. BET, XRD and TEM characterizations confirm that the MFA-derived TiO_2_ aerogel maintains physicochemical properties comparable to the conventional sample, and its photocatalytic reaction rate constant for tetracycline hydrochloride degradation is approximately twice that of the original sample.

## 1. Introduction

TiO_2_ aerogels are considered promising photocatalytic and photovoltaic materials due to their excellent photovoltaic properties, chemical stability, and high specific surface area [1,2]. In particular, their high surface area and large pore volume significantly enhance contaminant adsorption in practical applications [3,4]. The sol–gel technique has been extensively applied to prepare TiO_2_ aerogels [5,6]. The gel network formed after hydrolysis and condensation provides pores to hold the solvent, and the capillary forces can cause TiO_2_ gel structure shrinkage during drying, thus reducing the specific surface area of the gel [7,8,9]. Typical drying techniques for TiO_2_ aerogel preparation include supercritical drying [10], freeze drying [11], atmospheric pressure drying [12], and vacuum drying [13]. Additionally, traditional sol–gel methods generally require >40 h and large amounts of organic solvents for solvent exchange, hindering their applicability for large-scale industrial TiO_2_ aerogel preparation, and the large consumption of volatile organic solvents also brings potential environmental risks and increases production costs. These bottlenecks have become the main obstacle restricting the large-scale application of TiO_2_ aerogels in environmental remediation. Therefore, it is of great practical significance to develop a green, efficient and low-consumption preparation process.

**Figure 1:**
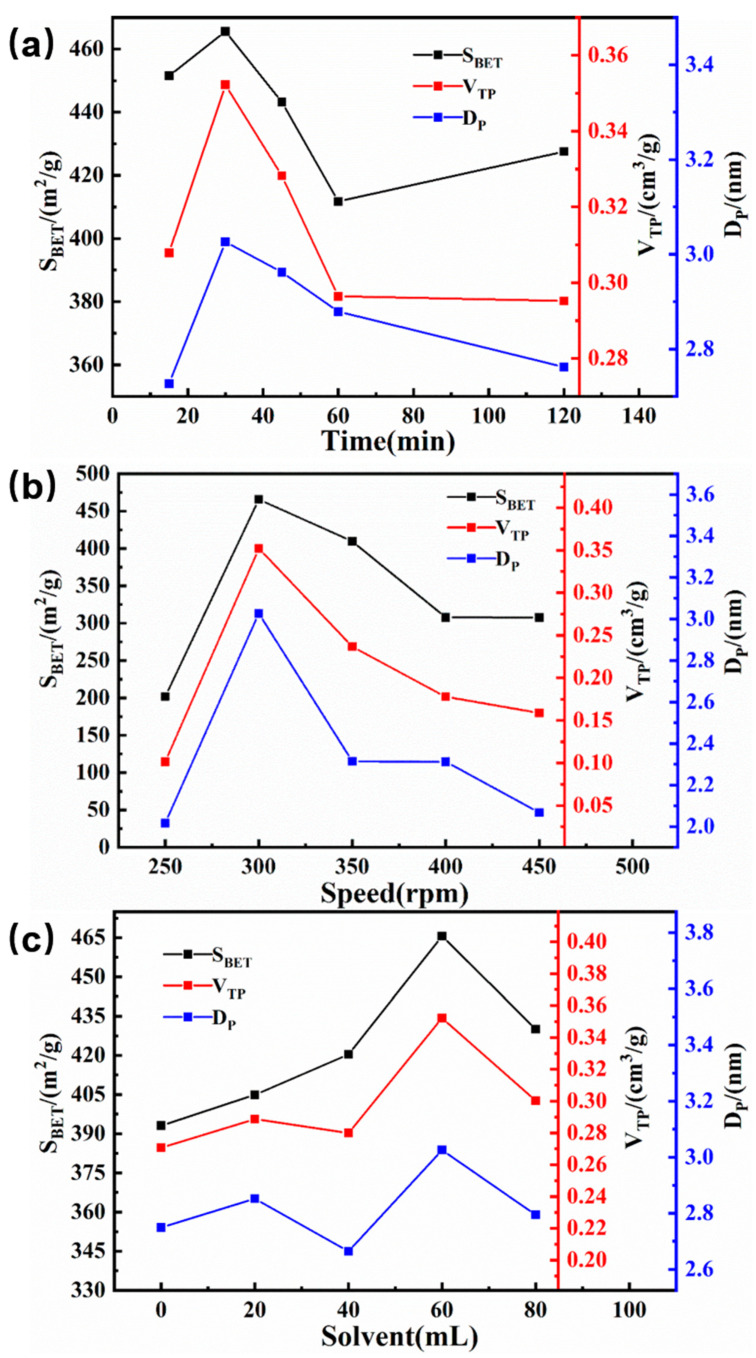
BET characterization results: (**a**–**c**) comparison of *S_BET_*, *V_TP_*, and *D_P_* with different milling times, milling speeds, and solvent consumption.

Mechanical milling, which introduces mechanical energy, proves a highly efficient and environmentally friendly method to reduce synthesis time and organic solvent consumption [14,15]. The structural and property aging of the gel involves neck growth and Oswald maturation mechanisms [15]. Mechanical milling promotes particle pulverization and growth by continuously exposing particle surfaces [16]. In addition, it facilitates excellent mass transfer as an agitation method [17], thus promoting solvent exchange. On this basis, mechanical milling is speculated to effectively facilitate gel aging and liquid–liquid exchange during solvent replacement, as shown in Figure 1a.

In recent years, extensive studies on TiO_2_ aerogels have focused on improving photocatalytic performance via elemental doping [18], heterojunction construction [19] and noble metal modification [20], and remarkable progress has been made in material property optimization. However, research on the greening and efficiency improvement of the TiO_2_ aerogel preparation process is still relatively limited [21]. Most preparation methods still rely on long-time static aging and multi-step solvent exchange, which cannot meet the demands of low-cost and large-scale production [20,21]. Moreover, conventional routes generally suffer from high equipment requirements, large volatile organic compound (VOC) emissions, and high operating costs, which further restrict their industrial translation. From the perspective of process intensification, introducing mechanical force into gel post-treatment is expected to break through these bottlenecks by improving mass transfer efficiency and reducing solvent and energy input simultaneously.

Differently from the above studies focusing on material performance modification, this work starts from preparation process optimization, and introduces mechanical ball milling into the gel aging and solvent exchange stage to achieve efficient and green synthesis of TiO_2_ aerogels.

To address the complex gel aging and solvent exchange processes, this study presents a novel and green synthesis method to rapidly prepare TiO_2_ aerogels in large quantities. Specifically, a simple ultrasound-assisted sol–gel process is used to prepare TiO_2_ gels, followed by mechanical milling to accelerate gel aging and solvent exchange. As clarified in the following sections, this work focuses on process optimization of TiO_2_ aerogel preparation: the MFA strategy achieves comparable material properties with greatly shortened preparation time and reduced solvent consumption. The proposed method is of great value for the large-scale production of TiO_2_ aerogels in industry.

## 2. Results

**Figure 2:**
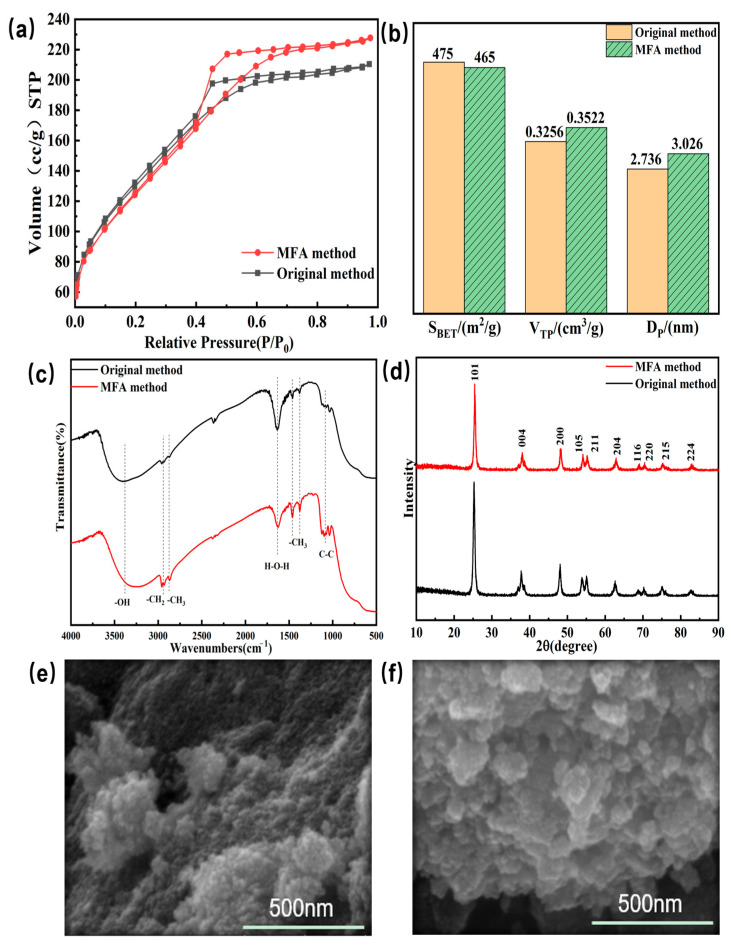
(**a**) N_2_ adsorption and desorption curves of TiO_2_ aerogels; (**b**) *S_BET_*, *V_TP_*, and *D_P_* for TiO_2_ aerogels; (**c**) FT-IR spectra of uncalcined TiO_2_ aerogels; (**d**) XRD spectrogram of TiO_2_ aerogels; (**e**) FESEM images of TiO_2_ aerogels prepared by the original method; (**f**) SEM images of TiO_2_ aerogels prepared by the MFA method.

The effects of milling time and solvent dosage on the specific surface area of TiO_2_ aerogels were investigated using the single-factor controlled variable method, as shown in Figure 1. The rationale for the parameter selection and screening process is provided in the Supplementary Information. The specific surface area of the TiO_2_ aerogel (*S_BET_*) peaked at a ball milling time of 30 min, a ball milling speed of 300 rpm, and a solvent consumption of 60 mL. The relationship between the milling time, milling speed, and solvent consumption with the specific surface area, total pore volume (*V_TP_*), and mean pore diameter (*D_P_*) of the TiO_2_ aerogel is shown in Figure 2. In addition, the *S_BET_*, *V_TP_*, and *D_P_* of TiO_2_ aerogels tended to increase and then decrease with increasing milling time, milling speed, and solvent consumption [22,23]. Adjusting the milling time and solvent consumption essentially alters the collision frequency of the reactants. Prolonged milling time contributes to complete solvent exchange and gel aging, whereas excessive milling time may cause gel particle agglomeration and disrupt the pore structure, thereby reducing the specific surface area [24]. As the solvent dosage increases, more of the high-surface-tension solvent can undergo exchange, and more of the pore structures can be retained in the subsequent drying process. Yet, excessive solvent reduces the energy utilization efficiency, thus canceling out the effect of milling. Single-factor optimization with SBET as the evaluation index was performed to screen preparation parameters (milling time, rotation speed, n-hexane dosage). All three parameters followed a volcano-type variation trend, and the optimal set of operating conditions at 30 min, 300 rpm and 60 mL was verified via replicate trials. Excessively long milling duration or high rotation speed cause particle agglomeration and pore collapse, whereas insufficient solvent results in incomplete solvent exchange and pore shrinkage during the drying stage.

**Figure 3:**
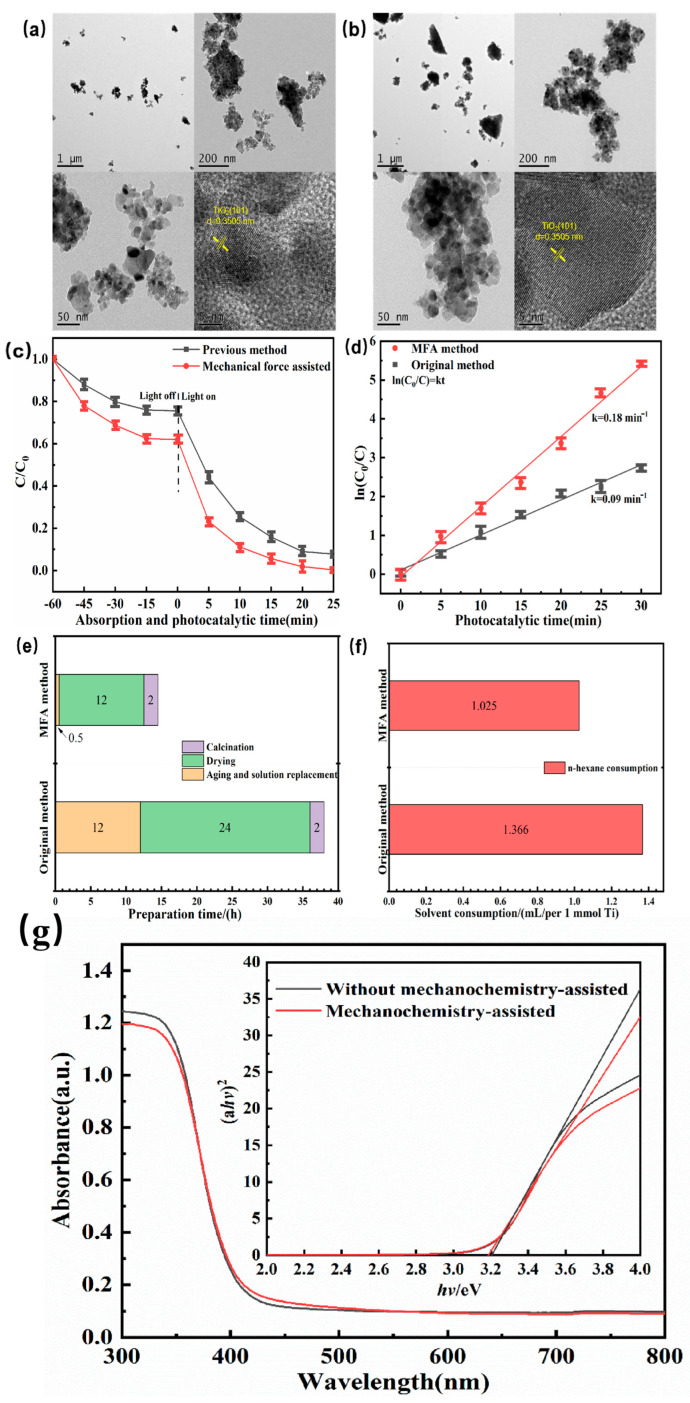
(**a**) TEM image of the sample prepared by the original method; (**b**) TEM image of the sample prepared by the MFA method; (**c**) adsorption and photocatalytic performance of TiO_2_ aerogels prepared by the original and MFA methods for TCH degradation; (**d**) pseudo-first-order kinetic fitting of TCH photodegradation; (**e**) comparison of total preparation times; (**f**) comparison of solvent consumption; (**g**) UV-Vis DRS spectra and corresponding Tauc plots of TiO_2_ aerogels prepared by different methods.

The BET characterization data in Figure 2a,b demonstrate that the specific surface area (*S_BET_*) of the TiO_2_ aerogels prepared using the two methods is similar, even though the total pore volume *V_TP_* and pore diameter *D_P_* of the samples prepared with the MFA method are slightly higher than those of the samples prepared by the original method [25,26]. According to the FT-IR spectra in Figure 2c, the samples prepared with the MFA method exhibit weaker absorption peaks attributed to adsorbed water H-O-H and stronger absorption peaks attributed to hydrocarbons compared to the samples prepared by the original method. Therefore, the solvent replacement effect of the MFA method was better than that of the original method [27]. Notably, the above textural and chemical structure differences between the two samples are slight. The MFA method is not designed to fabricate a substantially superior TiO_2_ aerogel, but to achieve comparable material properties with higher preparation efficiency and lower solvent consumption. More importantly, the optimized solvent exchange effect brings about improved pore connectivity and accessible pore volume, which cannot be fully reflected by the overall specific surface area value. Figure 2d shows the XRD spectra of the prepared TiO_2_ aerogels calcined at 500 °C for 2 h. According to the standard diffraction spectra (JCPDS No. 21-1272) [28], the TiO_2_ aerogels are pure anatase TiO_2_ [9,29]. Figure 3e,f present the FESEM results characterizing the morphological properties of TiO_2_ aerogels. Obviously, the TiO_2_ aerogel agglomerates prepared by the MFA method are larger with irregular porous layered surfaces, possibly attributed to the continuous mechanical forces promoting crystal growth [10,30].

To further reveal the surface chemical state and lattice defects of the MFA-derived TiO_2_ aerogel, XPS measurements were performed, and full spectral fitting results are presented in Figure S1 of the Supplementary Information. XPS results confirm that only Ti^4+^ exists in the sample without low-valent Ti signals, proving that mechanical ball milling does not generate oxygen vacancies or lattice defects; two oxygen species including lattice oxygen and surface hydroxyl oxygen are also distinguished from the O 1s spectrum. Figure 3a,b show that the TiO_2_ aerogels prepared by the two methods contain abundant pore structures, and the measured crystalline plane spacing is 0.3505 nm, corresponding to the anatase (101) crystalline plane of TiO_2_. Hence, the samples are pure anatase crystal structures with no lattice distortion [31]. The optical absorption properties of the as-prepared TiO_2_ aerogels were characterized by UV-Vis DRS, as shown in Figure 3. Both samples exhibit a typical absorption edge of anatase TiO_2_ at around 385 nm, and no obvious red shift or blue shift in the absorption edge is observed between the MFA and original samples.

The inset Tauc plot derived from the Kubelka–Munk function shows that the band gap energies of the original sample and the MFA sample are calculated to be 3.24 eV and 3.23 eV, respectively. The negligible difference in band gap indicates that mechanical force treatment does not alter the intrinsic electronic structure of TiO_2_ aerogels. This result further confirms that the improvement in photocatalytic performance is mainly attributed to the optimized pore structure, enhanced mass transfer efficiency and increased accessible active sites, rather than a change in band gap energy. As shown in Figure 3c,d, the TiO_2_ aerogel prepared by the MFA method demonstrates higher photocatalytic activity for TCH degradation relative to the aerogel prepared by the original method. All degradation curves are presented with error bars from three independent parallel experiments, and both fit well with the pseudo-first-order reaction kinetics. The pseudo-first-order reaction rate constant of the MFA sample (0.18 ± 0.011 min^−1^) is approximately twice that of the original sample (0.09 ± 0.007 min^−1^). Student’s t-test analysis confirms that the performance difference between the two samples is statistically significant (*p* < 0.05), demonstrating the reliability of the performance improvement trend. Cyclic degradation tests over five runs (Figure S2 in Supplementary Information) verify the favorable reusability of MFA-derived TiO_2_ aerogel. The removal efficiency only slightly declines from 95.63% to 90.44% and remains above 89% after five cycles, indicating outstanding structural stability during repeated photocatalysis. Notably, the modest difference in bulk specific surface area cannot fully reflect the change in accessible catalytic active sites. The increased total pore volume and average pore size of the MFA sample optimize the pore size distribution and enhance pore connectivity, which effectively reduces the mass transfer resistance of TCH molecules inside the pore channels. For conventional TiO_2_ aerogels with a high proportion of micropores, most internal active sites are difficult to access by pollutant molecules due to diffusion limitations. In contrast, the MFA treatment enlarges the pore size and shortens the diffusion path, greatly improving the utilization efficiency of internal active sites. Furthermore, the mechanical shearing effect during ball milling refines gel particles and exposes more reactive crystal facets on the material surface, increasing the density of effective surface active sites. These combined effects of optimized mass transfer and increased accessible active sites account for the significant improvement in photocatalytic reaction rate, despite the slight difference in overall specific surface area.

Based on the classic anatase TiO_2_ photocatalytic system, the primary reactive oxygen species driving TCH degradation can be inferred as superoxide radicals (·O_2_^−^) generated by the reduction of dissolved oxygen via photogenerated electrons, and hydroxyl radicals (·OH) formed by the oxidation of surface hydroxyl groups or water molecules via photogenerated holes [32,33]. The overall reaction follows a typical pathway: pollutant molecules are first adsorbed and enriched on the porous surface, then photogenerated electron–hole pairs are excited under UV irradiation, and finally the migrated surface active species oxidize the target pollutants into small molecules. It should be noted that in-depth mechanism verification via radical trapping experiments and electron paramagnetic resonance (EPR) characterization is not the core focus of this work, which prioritizes the development of the novel preparation strategy.

It is worth noting that the enhanced photocatalytic performance brought by the MFA treatment is not limited to tetracycline hydrochloride. As a typical anatase TiO_2_ aerogel with an optimized pore structure, the prepared material possesses intrinsic photocatalytic activity toward a broad spectrum of organic pollutants, including dyes (e.g., methyl orange and rhodamine B), phenolic compounds (e.g., p-nitrophenol) and other refractory organic contaminants. The optimized pore size distribution and improved mass transfer efficiency are universally beneficial for the adsorption and degradation of organic molecules with different molecular sizes and structural characteristics, indicating the broad application prospect of this MFA-derived TiO_2_ aerogel in water environment remediation.

Benefiting from the pure anatase crystal phase, intact porous framework and intrinsic chemical inertness of TiO_2_, the as-prepared MFA-derived aerogel possesses good structural stability in conventional aqueous photocatalytic systems. The well-crystallized anatase phase and robust three-dimensional network structure endow the material with basic resistance to particle agglomeration and structure collapse during repeated use, indicating favorable application potential in practical wastewater treatment. Systematic cycling experiments and long-term stability tests will be performed in our follow-up engineering application research to comprehensively evaluate the reusability of the material.

Figure 3e,f compare the time and solvent consumption between the original method and the MFA method, where the solvent consumption is the calculated amount of n-hexane used per mole of Ti. The MFA operation significantly shortens the time for solvent exchange from 12 h to 0.5 h, which improves the solvent exchange efficiency while preserving the high surface area of the TiO_2_ aerogel. Compared with the original method, the total time cost of the MFA method is reduced by about 60%, and the n-hexane consumption is reduced by 25%. Table 1 compares the MFA method with previously reported TiO_2_ aerogel synthesis routes. As summarized in the table, most reported synthesis routes rely on long-time static aging and multi-step solvent exchange, suffering from low production efficiency and high solvent consumption. Although some works achieve higher specific surface areas, the MFA method stands out for its ultra-short preparation cycle, low solvent usage, and superior scalability. Unlike supercritical drying routes that require high-pressure equipment and strict operating conditions, mechanical ball milling is a mature industrial technology with widely available facilities, which greatly lowers the threshold for large-scale production. From an environmental perspective, the 25% reduction in n-hexane consumption directly decreases VOC emissions and wastewater treatment load, aligning with green chemistry principles.

Preliminary quantitative analysis further demonstrates the economic advantages of the MFA strategy. In terms of energy consumption, the conventional solvent exchange process requires continuous heating at 45 °C for 12 h in a water bath oscillator, while the MFA method only requires 30 min of ball milling operation, reducing the energy consumption of the aging and solvent exchange stage by approximately 70% per batch. Overall, the comprehensive production cost per batch is estimated to be reduced by about 30% compared with the conventional method, and the cost advantage will be further amplified in scale-up production. Ball milling technology has mature industrial-scale equipment and a wide application foundation in material processing, so the MFA method has good scale-up feasibility.

## 3. Materials and Methods

### 3.1. Materials

The reagents include tetrabutyl titanate (C_16_H_36_O_4_Ti, purity ≥ 99.5%, CP, Sinopharm Chemical Reagent Co., Ltd., Shanghai, China), ethanol (C_2_H_5_OH, purity ≥ 99.7%, AR, Chengdu Kelong Chemical Industry Co., Ltd., Chengdu, China), n-hexane (CH_3_(CH_2_)_4_CH_3_, purity ≥ 99.7%, AR, Guangdong Guanghua Sci-Tech Co., Ltd., Shantou, China), deionized water, and 1 mol/L HCl. All chemical reagents were used as received.

### 3.2. TiO_2_ Aerogel Synthesis

**Figure 4:**
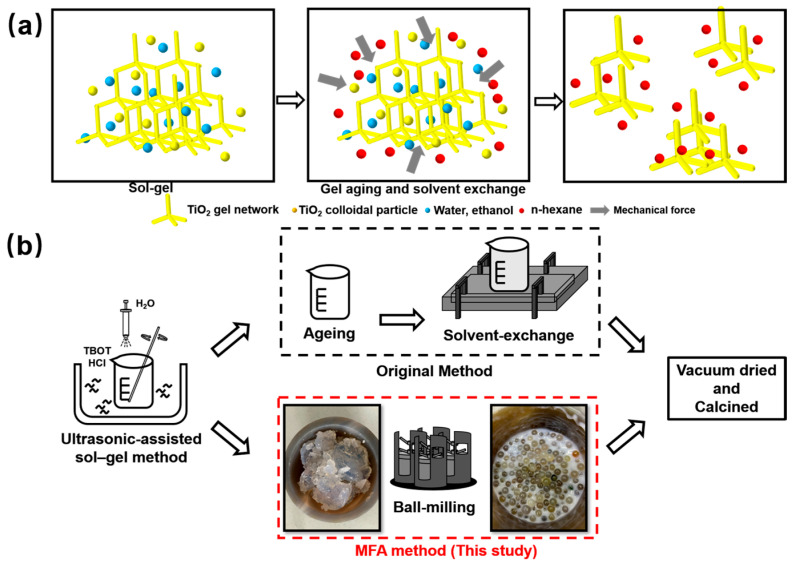
(**a**) Mechanisms of TiO_2_ aerogel preparation using the MFA method. (**b**) Comparison of the proposed preparation method with the original method.

Two different synthesis routes were employed to prepare TiO_2_ gels: the previously reported method [38] (original method) and the improved method based on the mechanical force-assisted method (MFA method; Figure 4).

With the original method, ethanol (20 mL) and 1 mol/L HCl (0.5 mL) were mixed in a 100 mL beaker under ultrasonication in an ultrasonic cleaner for 1 min. Then, TBOT (5 mL) was added. Next, 3.4 mL of deionized water was quickly sprinkled into the beaker with a sprinkler developed by our research team, and alcogel formation was allowed for 10 s. The alcogel was immersed in n-hexane and then wrapped well in plastic. Then, the samples were placed in a constant oscillating temperature bath at 45 °C for 12 h, followed by drying in a vacuum drying oven at 60 °C for 24 h. With the MFA method, the gel was formed the same way. The 500 mL agate jar was paired with 6 mm agate grinding balls, with a ball-to-wet-gel mass ratio of 3.7:1. A forward–pause–reverse cycle (5 min each) was applied to achieve homogeneous mixing and avoid local overheating. The total filling volume of gel, balls and solvent accounted for ~70% of the jar. Ethanol induced gel collapse while the blend demanded a long exchange time and delivered low *S_BET_* near 350 m^2^·g^−1^. N-hexane was chosen for low surface tension, high volatility and good gel compatibility to relieve vacuum drying capillary stress. Afterward, the slurry was filtered out using a nylon filter and dried at 60 °C in a vacuum drying oven for 12 h. Milling time and solvent consumption optimization processes are shown in Figure 1. The aerogel samples were calcined at 500 °C for 2 h.

### 3.3. Characterization

Nitrogen adsorption–desorption isotherms were measured at 77 K on a Quantachrome NOVA4200E nitrogen adsorption instrument, Quantachrome Instruments, Boynton Beach, FL, USA. TiO_2_ aerogel samples were pre-degassed under vacuum at 300 °C for 3 h before testing. Crystalline structures were characterized by a Dandong Haoyuan X-ray diffractometer with Cu Kα radiation (*λ* = 1.5406 Å) operating at 40 kV and 30 mA. Surface morphologies were observed using a HITACHI SU8020 field-emission scanning electron microscope. UV-Vis diffuse reflectance spectra (UV-Vis DRS) were acquired on a SHIMADZU UV-2550 spectrophotometer from 300 to 800 nm with BaSO_4_ as the reference, and the band gap energy (*Eg*) was calculated via the Kubelka–Munk-based Tauc plot method. Surface elemental composition and chemical valence states of calcined TiO_2_ aerogels were analyzed by an Escalab 250xi X-ray photoelectron spectroscopy (XPS) spectrometer (Thermo Fisher Scientific, East Grinstead, West Sussex, UK), equipped with Al Kα monochromatic X-ray source operated at 12 kV and 6 mA. Ball milling experiments were carried out on a QM-3SP2 planetary ball mill (Nanjing University Instrument Factory, Nanjing, Jiangsu Province, China).

### 3.4. Photoreactivity Measurement

Tetracycline hydrochloride (TCH, 50 mL, 10 mg/L) photodegradation tests were conducted in a 100 mL cylindrical quartz reactor (inner diameter: 40 mm; height: 80 mm) under the irradiation of a 300 W high-pressure mercury lamp (main emission wavelength: 365 nm) to determine the photocatalytic activity of the TiO_2_ aerogels [31]. The vertical distance between the light source and the liquid surface was 15 cm, and the light intensity at the liquid surface was calibrated to 35 mW/cm^2^ using an ultraviolet radiometer.

Before the photocatalytic reaction, the TCH solution and 0.025 g of TiO_2_ aerogel catalyst were mixed, and the suspension was magnetically stirred continuously in the dark for 1 h to achieve adsorption–desorption equilibrium. During the reaction, 2 mL of suspension was sampled at 0, 5, 10, 15, 20, 25 and 30 min, and filtered through a 0.45 μm nylon syringe filter before measurement. The TCH concentrations were measured using a UV-2550 UV–vis spectrophotometer (Shimadzu Corporation, Kyoto, Japan) at the characteristic wavelength of 357.5 nm.

All photocatalytic degradation experiments were performed in three independent parallel runs to ensure reproducibility. The kinetic parameters are expressed as mean ± standard deviation. The statistical significance of the performance difference between the original and MFA samples was analyzed by Student’s *t*-test, with *p* < 0.05 considered statistically significant.

## 4. Conclusions

This work develops a mechanical force-assisted (MFA) strategy for the green and ultrafast preparation of TiO_2_ aerogels, with process intensification as its core scientific contribution. For the first time, mechanical ball milling is introduced into the gel aging and solvent exchange stages of TiO_2_ aerogel synthesis, which significantly shortens the total preparation time by 60% and reduces n-hexane consumption by 25% while maintaining comparable physicochemical properties of the material. This work reveals the regulation mechanism of mechanical force on the pore structure of TiO_2_ aerogels, providing a new approach for the efficient and green synthesis of aerogel materials. Practically, the proposed method requires no specialized equipment and is compatible with existing industrial ball milling facilities, offering a feasible route for the low-cost, large-scale production of TiO_2_ aerogels. The MFA-derived aerogel also exhibits enhanced photocatalytic activity with a reaction rate constant approximately twice that of the conventional sample, further verifying the application value of this green preparation strategy. Nevertheless, this work mainly focuses on the process innovation of rapid preparation, and certain limitations remain. The effects of solution pH, initial pollutant concentration and reaction temperature on the interfacial photocatalytic process have not been systematically investigated, and the long-term cycling stability under continuous operation still needs further verification. Building on the established preparation strategy, our follow-up research will carry out systematic condition optimization, stability evaluation, mechanistic investigation, and comprehensive degradation performance verification toward various representative pollutants to further promote the practical application of this material in environmental remediation. Overall, the proposed MFA strategy holds great prospects for the industrial-scale production of high-performance TiO_2_ aerogels.

## Figures and Tables

**Table 1:** Comparison of preparation efficiency, solvent consumption and textural properties of TiO_2_ aerogels via different preparation methods.

Preparation Method	Drying Method	Aging Time	Solvent Exchange Time	Solvent Consumption (per 1 mmol Ti)	Highest *S_BET_* (m^2^/g)	References
Sol–gel method	Supercritical drying	~12 h	~24 h	Unspecified	602	[34]
Nanoparticle gelation method	Supercritical CO_2_ drying	≥30 min	~24 h	Unspecified	550	[2]
Sol–gel method	Ambient pressure drying	12 h	48 h	Unspecified	486	[35]
Metal-doped sol–gel method	Supercritical CO_2_ drying	~12 h	120 h	Unspecified	160	[36]
Ultrasonic-assisted sol–gel	Vacuum drying	>3 h	12 h	1.366 mL n-hexane	563	[37]
Ultrasonic-assisted sol–gel with mechanical force assistance	Vacuum drying	30 min		1.025 mL n-hexane	465	This study

## Data Availability

Data are contained within the article.

## Supplementary Materials

The following supporting information can be downloaded at: https://www.mdpi.com/article/10.3390/molecules31152690/s1, Figure S1: XPS spectra of TiO_2_ aerogels calcined at 500°C, (a) full sweep spectrum, (b) Ti 2p, (c) O; Figure S2:Cyclic photocatalytic degradation performance of tetracycline hydrochloride (TC-HCl).

## References

[1] Kiwic D., Tervoort E., Thach Y.V., Niederberger M. (2025). Mild Synthesis of Ag-Decorated TiO_2_ Aerogels for Light-Driven CO_2_ Reduction. ACS Mater. AU.

[2] Matter F., Kiwic D., Bernet M., Tervoort E., Niederberger M. (2025). Shaping nanoparticle-based aerogels for efficient light-driven catalysis. J. Mater. Chem. A.

[3] Rebber M., Sannemüller H., Jaruszewski M., Pfannkuche D., Urakawa A., Koziej D. (2023). Light and Mass Transport Computations Guide the Fabrication of 3D-Structured TiO_2_ and Au/TiO_2_ Aerogel Photocatalysts for Efficient Hydrogen Production in the Gas Phase. Chem. Mater..

[4] Son J.Y., Jo Y., Lee H., Jang Y.J., Kim H. (2025). Water hyacinth-inspired self-floating photocatalytic system for efficient and sustainable water purification. npj Clean Water.

[5] Protyai M.I.H., Saif S.J.A., Rahman M.M., Mural H., Rashid A.B. (2026). An Overview of Recent Progress of Green Nano-Composites for Sustainable Energy Storage Applications. Eng. Rep..

[6] Zhao H., Lei Y. (2020). 3D Nanostructures for the Next Generation of High-Performance Nanodevices for Electrochemical Energy Conversion and Storage. Adv. Energy Mater..

[7] Sun F., Yue X., Yu X., Di Y., Chen H., Xie S., Han W., Xi X., Zhang W., Zou H. (2025). The Effect of the Pore Size of TiO_2_ Aerogel on the Photocatalytic Decomposition of Formaldehyde. Catalysts.

[8] Guzel Kaya G., Yilmaz E., Deveci H. (2020). Synthesis of sustainable silica xerogels/aerogels using inexpensive steel slag and bean pod ash: A comparison study. Adv. Powder Technol..

[9] Jin R., Zhou Z., Liu J., Shi B., Zhou N., Wang X., Jia X., Guo D., Xu B. (2023). Aerogels for Thermal Protection and Their Application in Aerospace. Gels.

[10] Tinoco Navarro L.K., Jaroslav C. (2023). Enhancing Photocatalytic Properties of TiO_2_ Photocatalyst and Heterojunctions: A Comprehensive Review of the Impact of Biphasic Systems in Aerogels and Xerogels Synthesis, Methods, and Mechanisms for Environmental Applications. Gels.

[11] Zhang J., Zhang X., Tian Y., Zhong T., Liu F. (2021). Novel and wet-resilient cellulose nanofiber cryogels with tunable porosity and improved mechanical strength for methyl orange dyes removal. J. Hazard. Mater..

[12] Tao B., Qian Z., Miao F., Zhang P. (2025). Preparation and characterization of high-efficiency plasma dye-sensitized cell photoanode based on Cu/CuO/TiO_2_ ternary composite material. Microchim. Acta.

[13] Liao J., Yang J., Zhao J., Zhang J., Liu Q., Yan J. (2026). Electronic structure and optical anisotropy of N, B, La, and V-single doped TiO_2_. Ceram. Int..

[14] Kresse J., Uhlmann L., Christiansen A., Hübner R., Eychmüller A. (2025). Mortar Synthesis: A Mechanochemical Approach to Metal Aerogel Production. Chem. Mater..

[15] Jakupec N., Uran E., Jabłońska M., Užarević K., Palčić A. (2026). Thermo-mechanochemical interzeolite conversion in water-deficient systems. Microporous Mesoporous Mater..

[16] Hafner M.R., Juraj N.P., Flint K., Wiltsche H., Wolinski H., Amenitsch H., Doonan C.J., Užarević K., Carraro F. (2025). Vapor-Assisted Mechanochemical Synthesis of Enzyme and Hydrogen-Bonded Organic Framework Biocomposites. Small.

[17] Urata S., Kuo A.-T., Murofushi H. (2019). Relation between Microstructure and Flexibility of Doubly Cross-Linked Organic–Inorganic Aerogels. ACS Appl. Polym. Mater..

[18] Tang Q., Zhang Z., Pan Y., Leung M.K., Zhang Y., Chen K. (2025). Carbon Nitride Gels: Synthesis, Modification, and Water Decontamination Applications. Gels.

[19] Liu L., Xue Z., Sun Y., Wu Y. (2025). Photocatalytic properties of TiO_2_ nanofiber membranes co-modified with Ag and CuO. J. Alloys Compd..

[20] Tinello S., Glumac H., Niederberger M. (2026). Chiral TiO_2_-Based Aerogels from Ligand-Imprinted Nanoparticles: Implications for Heterogeneous Asymmetric Photocatalysis. ACS Appl. Nano Mater..

[21] Xu T., Donà L., Tan J.-C. (2026). Tuning of Electron-Donating Metal–Organic Frameworks toward High-Performance Triboelectric Nanogenerators for Self-Powered Shear Sensing. ACS Appl. Mater. Interfaces.

[22] Huang Z., Zhang Z., Han Y., Rong L., Yao H., Li C., Wang X., Mei T. (2026). Engineering TiO_2_/ZnS@MXene three-phase heterostructure for enhanced polysulfide capture and sulfur kinetics in high-performance lithium-sulfur batteries. J. Colloid Interface Sci..

[23] Sarvalkar P.D., Jagtap A.S., Vadanagekar A.S., Kamble S.S., Tibe A.P., Sheikh A.D., Vhatkar R.S., Sharma K.K.K. (2024). Multifunctional chitosan tailored γ-aluminum oxy-hydroxide monolith aerogels for sustained environmental remediation. Environ. Sci. Water Res. Technol..

[24] Sun C., Wang Z., Li X., Luo Y., Zheng H., Li X., Chen L., Li F. (2025). Adsorption–photocatalysis synergistic removal of perfluoroalkyl/polyfluoroalkyl substances by reusable chitin/polyethylene imine/O-g-C_3_N_4_ sponges in water environments. Chem. Eng. J..

[25] Kumar A., Thakur M.K., Hart P., Thakur V.K. (2026). Mechanistic Insights and Design Strategies for Hydrogel/Aerogel Sorbents in Remediation of Per- and Polyfluoroalkyl Substances. ACS Environ. AU.

[26] Bi Y., Meng X., Tan Z., Geng Q., Peng J., Yong Q., Sun X., Guo M., Wang X. (2024). A novel ZIF-L/PEI thin film nanocomposite membrane for removing perfluoroalkyl substances (PFASs) from water: Enhanced retention and high flux. Sci. Total Environ..

[27] Zhang K., Cheng P., Liu Y., Xia S. (2024). Efficient removal of per- and polyfluoroalkyl substances by a metal-organic framework membrane with high selectivity and stability. Water Res..

[28] Maheshwary P.B., Handa C.C., Nemade K.R., Chaudhary S.R. (2020). Role of nanoparticle shape in enhancing the thermal conductivity of nanofluids. Mater. Today Proc..

[29] Luo Y., Wang Y., Liao Y., Qiang R., Yuan Y., Ma Y., Sun X., Xu H., Zhang E., Li Y. (2025). TiO_2_ nanowire aerogels with interlocking network structure: Achieving ultra-low density and superior strength for thermal insulation. Chem. Eng. J..

[30] Zambianchi M., Khaliha S., Bianchi A., Tunioli F., Kovtun A., Navacchia M.L., Salatino A., Xia Z., Briñas E., Vázquez E. (2022). Graphene oxide-polysulfone hollow fibers membranes with synergic ultrafiltration and adsorption for enhanced drinking water treatment. J. Membr. Sci..

[31] Cao P., Liu Y., Zhang Q., Guo C., Li J., Zeng Q. (2025). Advanced Self-Bias Photocatalytic System for U(VI) and Tetracycline Hydrochloride Removal Coupled with Electricity Generation via Ti_3_C_2_@Cu1.96S/TiO_2_-Si Heterojunction. Adv. Funct. Mater..

[32] Zhang B., Sun B., Liu F., Gao T., Zhou G. (2024). TiO_2_-based S-scheme photocatalysts for solar energy conversion and environmental remediation. Sci. China Mater..

[33] Li Y., Yu B., Hu Z., Wang H. (2022). Construction of direct Z-scheme SnS2@ZnIn2S4@kaolinite heterostructure photocatalyst for efficient photocatalytic degradation of tetracycline hydrochloride. Chem. Eng. J..

[34] Matter F., Niederberger M. (2023). Optimization of Mass and Light Transport in Nanoparticle-Based Titania Aerogels. Chem. Mater..

[35] Zou W., Bian H., Guo J., Xu J., Guo B. (2023). Preparation of Titania–Silica Composite Aerogel at Atmospheric Pressure and Its Catalytic Performance in the Synthesis of Poly (Butylene Succinate). Materials.

[36] Lizeth Katherine T.N., Vendula B., Jaroslav K., Jaroslav C. (2023). Structure and Photocatalytic Properties of Ni-, Co-, Cu-, and Fe-Doped TiO_2_ Aerogels. Gels.

[37] Feng Q., Cai H., Lin H., Qin S., Liu Z., Ma D., Ye Y. (2018). Synthesis and structural characteristics of high surface area TiO_2_ aerogels by ultrasonic-assisted sol–gel method. Nanotechnology.

[38] Karaca M., Eroğlu Z., Açışlı Ö., Metin Ö., Karaca S. (2023). Boosting Tetracycline Degradation with an S-Scheme Heterojunction of N-Doped Carbon Quantum Dots-Decorated TiO_2_. ACS Omega.

