# Supplementary material for: Green and Ultrafast Preparation of TiO2 Aerogel Assisted by Mechanical Ball Milling
Source: Molecules. 2026 Aug 2;31(15):2690. doi: 10.3390/molecules31152690 (PMC13468275; doi:10.3390/molecules31152690)
Supplement: Supplementary file 1 [file molecules-31-02690-s001.zip › molecules-4429688-supplementary.pdf]

Supporting Information

Green and Ultrafast Preparation of TiO_2 Aerogel Assisted by Mechanical Ball Milling

Nai Liu [1,†], Jingyi Chen [2,†], Dongzhi Zhu [2], Kao Chen [2,3,*] and Qingge Feng [1,*]

[1] School of Resources, Environment and Materials, Guangxi University, Nanning 530004, China; liunai_96@163.com
[2] School of Materials and Environment, Guangxi Key Laboratory of Advanced Structural Materials and Carbon Neutralization, Guangxi Engineering Research Center for Advanced Materials and Intelligent Manufacturing, Guangxi Minzu University, Nanning 530105, China; cjy028888@163.com (J.C.); 20230021@gxmzu.edu.cn (D.Z.)
[3] College of Civil Engineering and Architecture, Guangxi University, Nanning 530004, China
* Correspondence: ck@gxmzu.edu.cn (K.C.); fengqg@gxu.edu.cn (Q.F.)
† These authors contributed equally to this work.

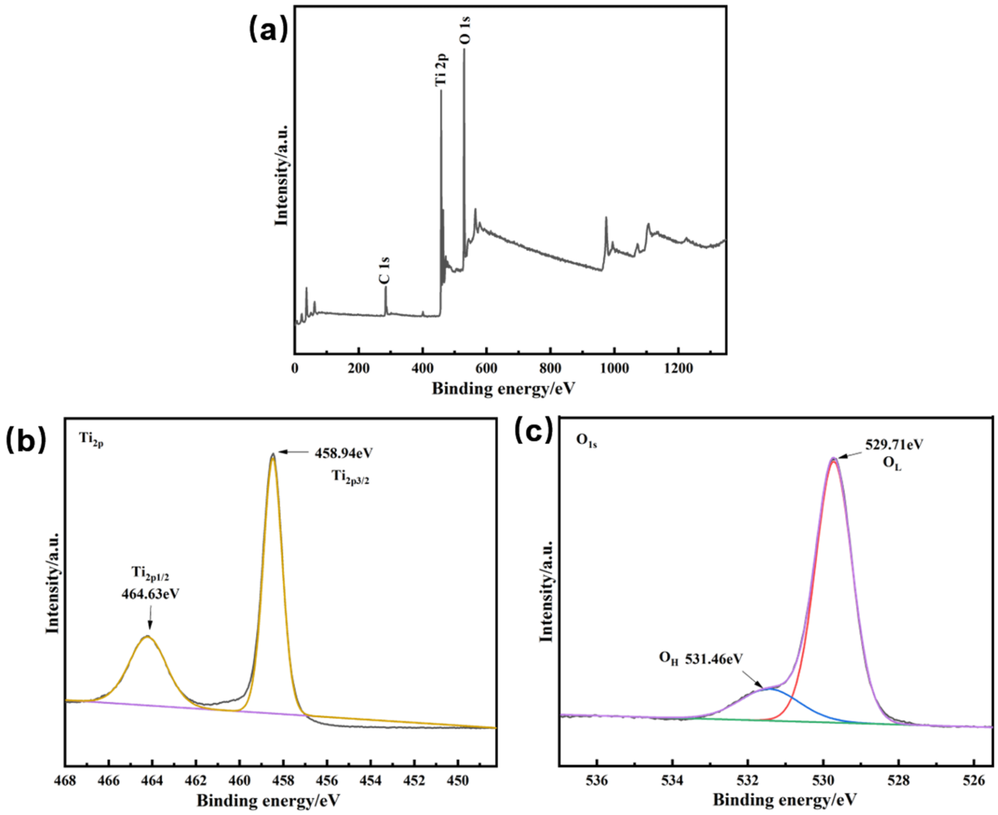

Figure. S1 XPS spectra of TiO_2 aerogels calcined at 500°C, (a) full sweep spectrum, (b) Ti 2p, (c) O.

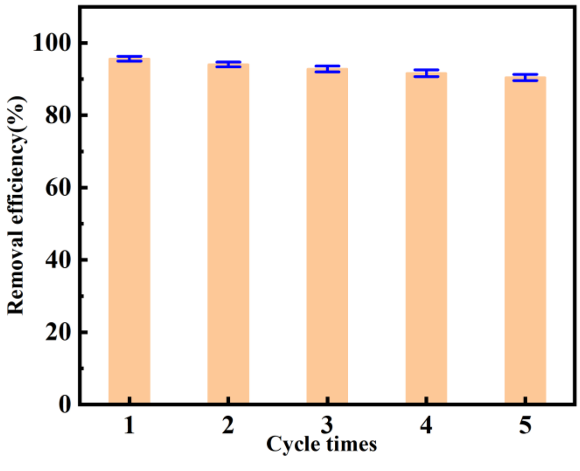

Figure. S2 Cyclic photocatalytic degradation performance of tetracycline hydrochloride (TC-HCl).
